# Prognosis and risk factors of chronic kidney disease progression in patients with diabetic kidney disease and non-diabetic kidney disease: a prospective cohort CKD-ROUTE study

**DOI:** 10.1080/0886022X.2022.2106872

**Published:** 2022-08-08

**Authors:** Shengnan Chen, Lei Chen, Hongli Jiang

**Affiliations:** Department of Blood Purification, Kidney Hospital, The First Affiliated Hospital of Xi'an Jiaotong University, Xi'an, China

**Keywords:** Diabetic kidney disease, non-diabetic kidney disease, 50% eGFR decline, kidney replacement therapy, all-cause death, CVD events

## Abstract

Diabetic kidney disease (DKD) is emerging rapidly as the leading cause of chronic kidney disease (CKD) worldwide. In this 3-year prospective, multicenter cohort study, a total of 1138 pre-dialysis CKD patients were recruited. Patients were categorized into two groups according to the etiologies of DKD and non-diabetic kidney disease (NDKD). Propensity score matching was performed to adjust for confounding factors, resulting in 197 patients being assigned to DKD and NDKD groups, respectively. The primary endpoints were 50% estimated glomerular filtration rate (eGFR) decline and initiation of kidney replacement therapy (KRT). The secondary endpoints were all-cause death and the development of cardiovascular disease (CVD) events. We found that DKD patients have a higher risk to develop 50% eGFR decline endpoint (HR:2.30, 95%CI [1.48–3.58], *p* < 0.001) and KRT endpoint (HR:1.64, 95%CI [1.13–2.37], *p* < 0.05) than NDKD patients. The 3-year cumulative incidence of 50% eGFR decline and KRT endpoint was significantly higher in DKD patients (26.90% *vs.*13.71% and 35.03% *vs.* 22.34%, respectively). The Cox regression analyses showed that the increased systolic blood pressure (SBP), DKD, decreased serum albumin (Alb), and higher CKD stages were risk factors for the 50% eGFR decline endpoint; the increased SBP, DKD, decreased serum Alb, serum creatinine (Scr), higher CKD stages, presence of proteinuria and CVD were risk factors for KRT endpoint; the increased age, decreased hemoglobin (Hb), decreased serum Alb were risk factors for all-cause death endpoint; the increased age, decreased serum Alb were risk factors for CVD events endpoint. Appropriate preventive or therapeutic interventions should be taken to control these predictive factors to delay the development of CKD complications, thereby improving the prognosis and reducing the disease burden of the high-risk populations.

## 1. Introduction

Chronic kidney disease (CKD) is a highly prevalent and increasingly serious public health problem worldwide [1]. A systematic analysis of the Global Burden of Disease Study showed that there are 697.5 million CKD patients worldwide and this accounts for 9.1% of the world’s population [2]. With an increasingly aging global population, the prevalence of diabetes is rapidly increasing [3]. Worldwide, 439 million and 783.2 million adults are estimated to develop diabetes by 2030 and 2045 respectively [4,5]. Therefore, the prevalence of diabetic kidney disease (DKD) is genuinely increasing in recent years and has become the leading cause of CKD worldwide [1]. Unlike other etiologies of CKD, both metabolic mechanisms, hemodynamic dysregulations, oxidative stress, immune and inflammatory response, autophagy [6], and so on are involved in the development and progression of DKD. Despite abundant therapeutic strategies have been made to manage diabetes, the prevalence of DKD remains high and has attracted a considerable amount of research in this area. However, few studies have been conducted to compare the prognosis of CKD patients according to the classification of DKD and non-diabetic kidney disease (NDKD).

Therefore, the present prospective cohort study aimed to further evaluate the differences in prognosis between DKD and NDKD patients. The primary objective of the present study was to assess the long‐term outcome of 50% estimated glomerular filtration rate (eGFR) decline and kidney replacement therapy (KRT) events between DKD and NDKD patients. And the secondary objective was to compare the outcome of all-cause death and cardiovascular disease (CVD) events in DKD and NDKD patients. In this way, it can be elucidated whether patients with DKD carry a worse prognosis than NDKD patients, and prognostic risk factors can also be explored. It is anticipated that our study may provide some preventive or therapeutic interventions for CKD patients by controlling these associated risk factors. At the same time, the disease burden of CKD patients can be reduced and the quality of life and prognosis of CKD patients can be improved through effective prevention.

## 2. Materials and methods

### 2.1. Data collection and study design

A 3-year prospective, observational, multicenter cohort study from the Chronic Kidney Disease Research of Outcomes in Treatment and Epidemiology (CKD-ROUTE) study was used for data analysis [7]. The design and baseline characteristics of the CKD-ROUTE study had been described in detail previously [7,8] and the data was kindly shared by original authors Soichiro Iimori *et al.* from the Dryad repository (DOI: 10.5061/dryad.kq23s). In conclusion, a total of 1138 CKD patients with stages 2–5 according to the Kidney Disease Improving Global Outcomes (KDIGO) classification who were not on dialysis from the Tokyo Medical and Dental University Hospital and its 15 affiliated hospitals in the Tokyo metropolitan area were recruited. Etiology of kidney disease in each patient was determined by the physician who was treating the patient at the time of enrollment, based on patients’ past histories, clinical characteristics and findings, and histological findings in biopsied kidney specimens [8]. Diabetes was defined as either HbA1c ≥ 6.5% or receiving anti-diabetic therapy [8]. Patients with a history of diabetes, loss of kidney function, presence of proteinuria, and pathological manifestations of DKD confirmed by kidney biopsy were defined as the DKD group. After excluding DKD, CKD patients who don’t have DKD were defined as the NDKD group including chronic glomerulonephritis, nephrosclerosis, and others [8]. Baseline characteristics of the participants were recorded at the initial medical examination, and the follow-up data were obtained every 6 months. eGFR was calculated using the modified three-variable Modification of Diet in Renal Disease (MDRD) equation developed by the Japanese Society of Nephrology [7–9]. This study was in accordance with the Declaration of Helsinki and approved by the ethics committees as described in the previous study [7,10]. And all the participants provided written consent [7,10].

### 2.2. Propensity score matching

In our present study, the participants were divided into two groups according to the etiologies of CKD. DKD patients were defined as the DKD group and other types of CKD were defined as the NDKD group. Propensity score matching (PSM) (1:1) was performed using a logistic regression model to reduce potential confounding factors including gender, age, systolic blood pressure (SBP), body mass index (BMI), hemoglobin (Hb), serum albumin (Alb), serum creatinine (Scr), eGFR, the prevalence of CVD, the prevalence of hypertension, CKD stage, proteinuria, urinary occult blood, use of renin-angiotensin system inhibitors (RASI), use of calcium channel blocker (CCB), and use of diuretics. Individual propensity scores were calculated and patients between DKD and NDKD groups were matched using the nearest-neighbor matching algorithm with a caliper value = 0.02. After PSM, 197 patients were assigned to each group respectively. Therefore, a total of 394 CKD patients were analyzed in our present study.

### 2.3. Endpoints

The primary endpoints in our study were composite kidney endpoints [11] including 50% eGFR decline (>50% eGFR loss) and initiation of KRT (including hemodialysis or peritoneal dialysis). The secondary endpoints were the all-cause death and development of CVD events. CVD events included ischemic heart disease, congestive heart failure, peripheral arterial disease, or stroke [7]. Patients were followed up until the occurrence of the endpoints or until the end of the cohort study.

### 2.4. Statistical analysis

For baseline characteristics, the independent Student’s *t*-test was used to compare normally distributed continuous variables between DKD and NDKD groups and presented as mean ± standard deviation ($\bar{x}\pm s$). Non-normally distributed continuous variables were compared using the Mann-Whitney U test and presented as medians and interquartile range (M, IQR). Categorical data were presented as frequencies and percentages and compared by the Chi-square test. Statistical analyses were performed using SPSS software, version 26.0.

Cumulative hazard curves of the primary and secondary endpoints were analyzed *via* the R packages ggplot2, survival, and survminer. The log-rank test was used to calculate the hazard ratio (HR), 95% confidence interval (CI), and P-value. Cox regression analyses were performed for the primary and secondary outcomes to further determine prognostic factors for 50% eGFR decline, KRT, all-cause death, and CVD events endpoints. The proportional hazard (PH) assumption was tested graphically through the Schoenfeld residual test for all variables using the cox.zph function in the survival R package. The variables that met the PH assumption were directly entered into the Cox proportional hazard models. The variables that didn’t meet the PH assumption were included in the model as time-dependent covariates (T_COV_variables: variables*ln(time)). Univariate Cox regression analyses were performed for all variables to calculate the *p*-values. Variables with a *p*-value <0.05 in the univariate Cox regression analyses were entered into multivariate Cox regression models to identify the independent risk factors for primary and secondary endpoints. All P values presented are based on two-tailed tests, and *p* < 0.05 was considered statistically significant.

## 3. Results

### 3.1. Baseline characteristics of the participants before and after PSM

A total of 1138 CKD patients were recruited in the CKD-ROUTE study, including 851 NDKD patients and 287 DKD patients. Before PSM, the baseline characteristics were significantly different between DKD and NDKD groups. After PSM, a total of 394 patients (197 pairs) remained for further analyses and the matched groups were well‐balanced. There were no statistically significant differences between the two groups after PSM (*p* > 0.05). Characteristics of the cohorts before and after PSM are presented in Table 1.

**Table 1.** Baseline characteristics between the DKD and NDKD groups before and after propensity score matching.

	Before‐PSM				After‐PSM			
	NDKD group	DKD group	*χ*^2^/*t*/*Z* value	*p*-Value	NDKD group	DKD group	*χ*^2^/*t*/*Z* value	*p*-Value
Number (*n*)	851	287	–	–	197	197	–	–
Gender (male/female, number)	589/262	203/84	0.234	0.629	145/52	149/48	0.214	0.643
Age (year, $\bar{x}$ ± *s*)	68.15 ± 14.05	66.07 ± 11.90	2.133	0.033	69.19 ± 13.39	66.97 ± 11.62	1.756	0.080
SBP (mmHg, $\bar{x}$ ± *s*)	137.93 ± 22.06	145.95 ± 23.09	−5.648	<0.001	143.91 ± 24.77	143.21 ± 23.05	0.291	0.772
BMI (kg/m^2^, $\bar{x}$ ± *s*)	23.25 ± 3.73	25.07 ± 4.57	−5.806	<0.001	24.79 ± 4.22	24.36 ± 3.99	1.054	0.292
Hb (g/L, $\bar{x}$ ± *s*)	121.85 ± 22.43	110.18 ± 21.18	7.966	<0.001	113.57 ± 22.21	112.50 ± 21.04	0.494	0.622
Serum Alb (g/L, $\bar{x}$ ± *s*)	39.61 ± 5.86	34.91 ± 6.64	10.603	<0.001	37.10 ± 6.61	36.43 ± 5.82	1.067	0.286
Scr (mg/dL, M, IQR)	1.59,1.31	2.36,1.84	167971.000	<0.001	2.17,1.91	2.20,1.83	20287.000	0.435
eGFR (ml/min/1.73m^2^, M, IQR)	33.03,29.74	20.18,20.11	78358.500	<0.001	23.67,16.42	21.44,15.41	18738.500	0.556
Prevalence of CVD (Yes, %)	194, 22.84	111, 38.70	27.585	<0.001	77,39.10	61,31.00	2.855	0.091
Prevalence of hypertension (Yes, %)	750, 88.10	277, 96.50	17.138	<0.001	194,98.50	187,94.90	3.898	0.048
CKD stage (2/3/4/5, number)	85/396/248/122	10/74/116/87	71.612	<0.001	10/54/79/54	7/54/86/50	0.980	0.806
Proteinuria (Yes, %)	458, 54.70	252, 88.40	103.924	<0.001	161,81.70	168,85.30	0.903	0.342
Urinary occult blood (Yes, %)	270, 32.30	108, 37.90	3.024	0.082	74,37.60	69,35.00	0.274	0.600
Use of RASI (Yes, %)	496, 58.30	224, 78.00	36.076	<0.001	154,78.20	147,74.60	0.690	0.406
Use of CCB (Yes, %)	374, 43.90	162, 56.40	13.454	<0.001	123,62.40	111,56.30	1.515	0.218
Use of diuretics (Yes, %)	232, 27.30	149, 51.90	58.576	<0.001	97,49.20	90,45.70	0.499	0.480

Abbreviations: DKD: diabetic kidney disease; NDKD: non-diabetic kidney disease; SBP: systolic blood pressure; BMI: body mass index; Hb: hemoglobin; Alb: albumin; Scr: serum creatinine; eGFR: estimated glomerular filtration rate; CVD: cardiovascular disease; CKD: chronic kidney disease; RASI: Renin-angiotensin system inhibitors; CCB: calcium channel blocker.

### 3.2. Comparison of the cumulative incidence of primary and secondary endpoints between DKD and NDKD groups in 3 years

The primary outcomes (50% eGFR decline and initiation of KRT) and secondary outcomes (all-cause death and CVD events) were defined as research endpoints in the present study. We calculated the 3-year cumulative incidence of endpoints and compared the differences in incidence between the DKD and NDKD groups. The results showed that the overall incidence of 50% eGFR decline, KRT, all-cause death, and CVD events were 20.30%, 28.68%, 10.15%, and 15.74%. The cumulative incidence of 50% eGFR decline, KRT, all-cause death, and CVD events for NDKD patients were 13.71%, 22.34%, 9.64%, and 13.71%. The cumulative incidence of 50% eGFR decline, KRT, all-cause death, and CVD events for DKD patients were 26.90%, 35.03%, 10.66%, and 17.77%. The incidence for primary endpoints was significantly higher in the DKD group than that in the NDKD group (*p* < 0.05). There were no significant differences in the incidence of secondary outcomes between the two groups (*p* > 0.05) (Table 2).

**Table 2.** Comparison of the cumulative incidence of primary and secondary endpoints.

	Overall (%)	NDKD (%)	DKD (%)	Chi-square value	*p* Value
Incidence of primary endpoints					
50% eGFR decline	20.30	13.71	26.90	10.603	0.001
KRT	28.68	22.34	35.03	7.755	0.005
Incidence of secondary endpoints					
All-cause death	10.15	9.64	10.66	0.111	0.739
CVD events	15.74	13.71	17.77	1.225	0.268

### 3.3. Cumulative hazard curves of endpoints

Cumulative hazard curves were produced to calculate the cumulative incidence and HR of primary outcomes (50% eGFR decline and initiation of KRT) and secondary outcomes (all-cause death and CVD events) between two groups. The results revealed that patients with DKD have a significantly higher risk to develop 50% eGFR decline endpoint than those NDKD patients (HR:2.30, 95% CI [1.48–3.58], *p* < 0.001) (Figure 1(A)). The risk of progression to KRT endpoint was much higher in the DKD group than NDKD group (HR:1.64, 95% CI [1.13–2.37], *p* < 0.05) (Figure 1(B)). And NDKD patients had a higher probability to be free from 50% eGFR decline endpoint and KRT endpoint (Figure 1(A,B)). No significant differences were observed regarding all-cause death and CVD events endpoints between the two groups (*p* > 0.05) (Figure 1(C,D)).

Figure 1.Cumulative hazard curves of endpoints. Primary endpoints: (A). 50% eGFR decline endpoint, (B). KRT endpoint. Secondary endpoints: (C). All-cause death endpoint, (D). CVD events endpoint.

**Figure:**
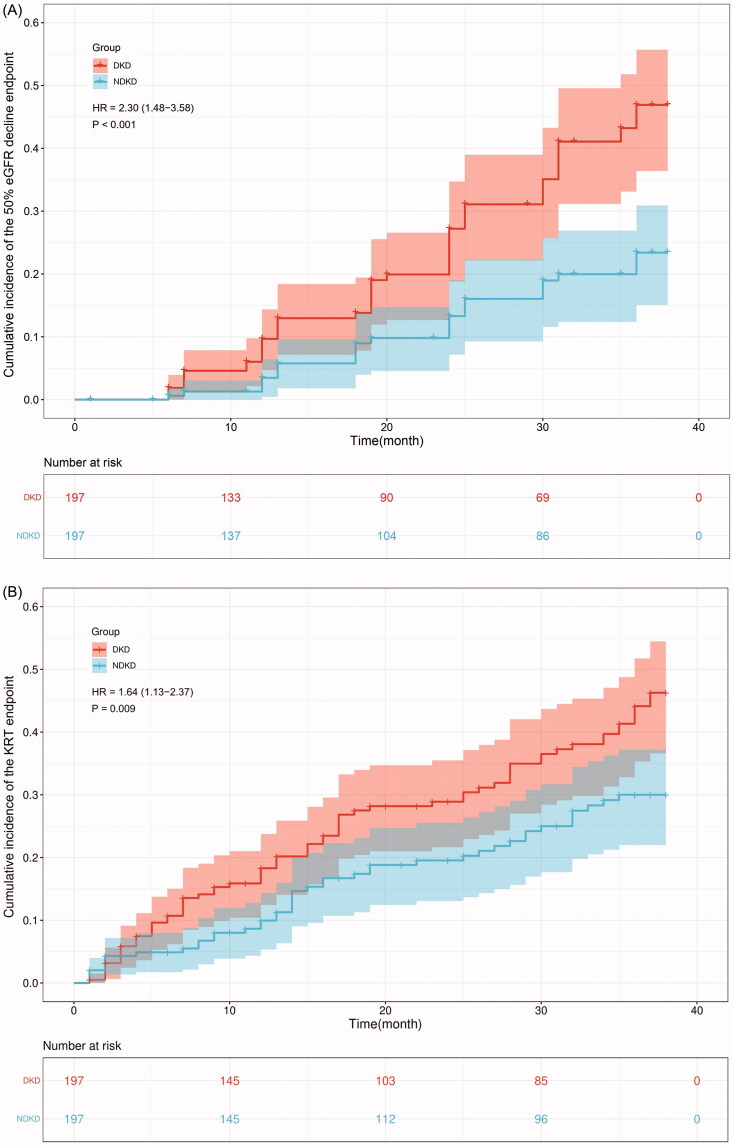

**Figure:**
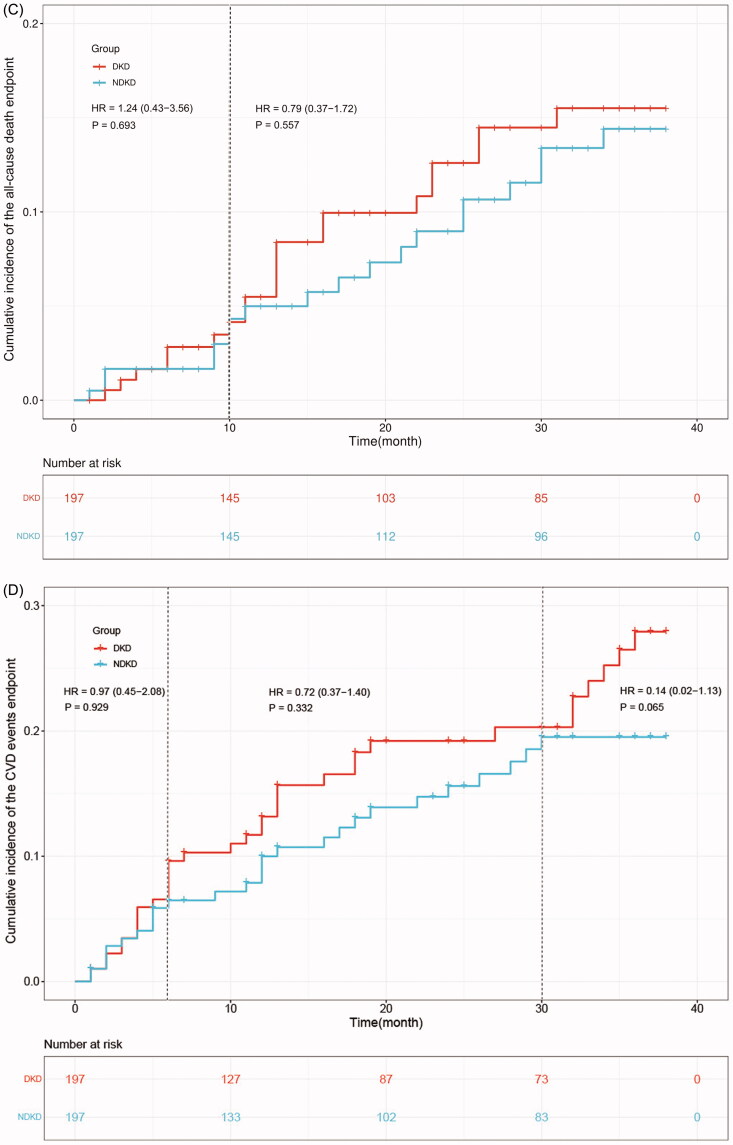

### 3.4. Prognostic factors for endpoints

The PH assumption tests were performed for variables in each endpoint and the results showed that all the variables associated with 50% eGFR decline endpoint, all-cause death endpoint, and CVD events endpoint met the PH assumption (*p* > 0.05). However, variables Scr and the prevalence of CVD violated the PH assumption in the KRT endpoint (*p* < 0.05). So, time-dependent covariates (T_COV_Scr and T_COV_ Prevalence of CVD) were constructed in the analysis of the KRT endpoint. The detailed Schoenfeld residual plots can be seen in the Supplementary Materials (Figure S1–S4).

**Figure 2.**
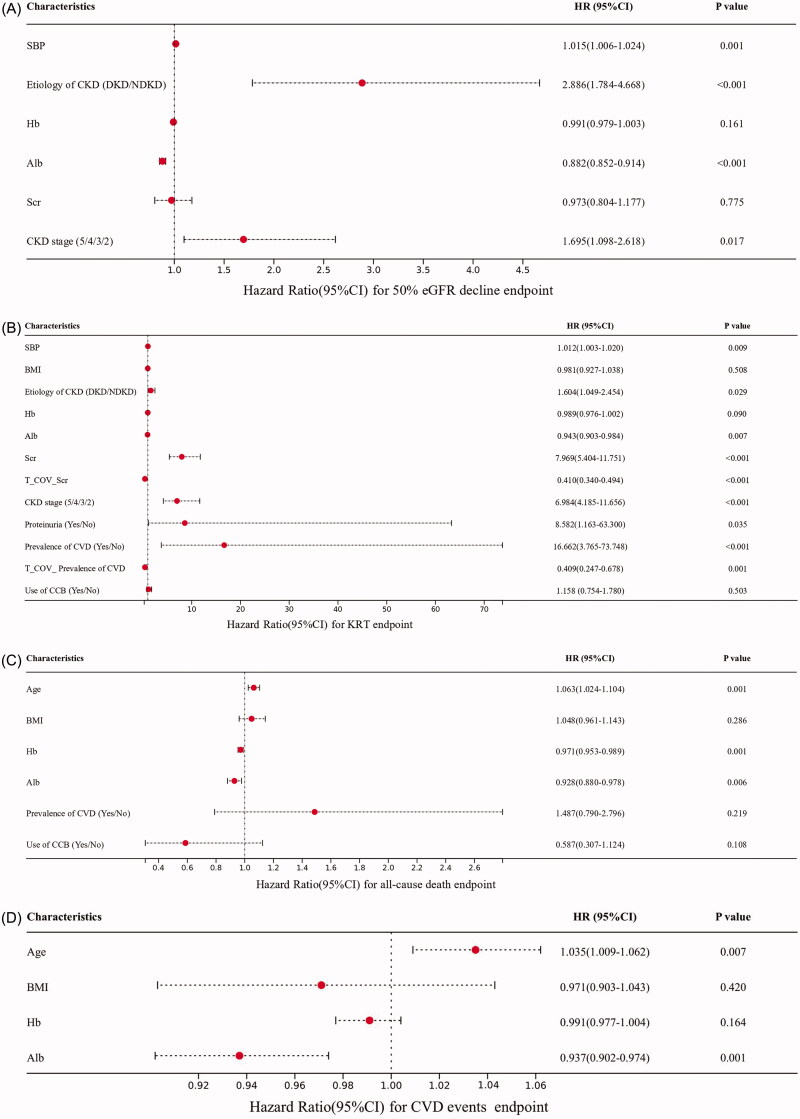
Multivariate Cox regression analyses for (A). 50% eGFR decline endpoint, (B). KRT endpoint, (C). All-cause death endpoint, (D). CVD events endpoint.

The results of the univariate Cox regression analyses for four endpoints can be seen in Table 3. The multivariate Cox regression analyses for 50% eGFR decline endpoint revealed that the increased SBP (HR:1.015, 95% CI [1.006–1.024], *p* < 0.05), DKD (HR for DKD *vs*. NDKD: 2.886, 95% CI [1.784–4.668], *p* < 0.001), decreased serum Alb (HR:0.882, 95% CI [0.852–0.914], *p* < 0.001), higher CKD stages (HR:1.695, 95% CI [1.098–2.618], *p* < 0.05) were independent risk factors for the progression of 50% eGFR decline endpoint (Figure 2(A)).

**Table 3.** Univariate Cox regression analyses for endpoints.

	50% eGFR decline endpoint		KRT endpoint		All-cause death endpoint		CVD events endpoint	
Characteristics	HR (95%CI)	*p* Value	HR (95%CI)	*p* Value	HR (95%CI)	*p* Value	HR (95%CI)	*p* Value
Gender (male/female)	1.465 (0.847-2.534)	0.172	0.862 (0.574-1.293)	0.472	0.852 (0.433-1.675)	0.642	1.239 (0.683-2.248)	0.481
Age	0.989 (0.972-1.007)	0.235	0.988 (0.973-1.002)	0.095	1.081 (1.041-1.122)	<0.001	1.043 (1.017-1.069)	0.001
SBP	1.012 (1.004-1.020)	0.004	1.015 (1.009-1.022)	<0.001	0.997 (0.983-1.010)	0.640	1.000 (0.990-1.011)	0.970
BMI	0.948 (0.894-1.006)	0.080	0.949 (0.903-0.997)	0.036	0.915 (0.840-0.996)	0.041	0.913 (0.853-0.978)	0.009
Etiology of CKD (DKD/NDKD)	2.340 (1.472-3.722)	<0.001	1.646 (1.127-2.403)	0.010	1.149 (0.618-2.138)	0.660	1.415 (0.856-2.338)	0.176
Hb	0.983 (0.972-0.993)	0.001	0.965 (0.956-0.974)	<0.001	0.964 (0.948-0.979)	<0.001	0.981 (0.968-0.993)	0.002
Serum Alb	0.906 (0.882-0.931)	<0.001	0.953 (0.927-0.979)	<0.001	0.921 (0.884-0.961)	<0.001	0.933 (0.901-0.966)	<0.001
Scr	1.142 (1.018-1.282)	0.023	1.780(1.574-2.012)	<0.001	1.128 (0.956-1.332)	0.153	1.105 (0.959-1.273)	0.167
T_COV_Scr	–	–	0.902 (0.855-0.952)	<0.001	–	–	–	–
CKD stage (5/4/3/2)	1.520 (1.130-2.044)	0.006	5.596 (4.049-7.734)	<0.001	1.393 (0.929-2.088)	0.109	1.290 (0.935-1.781)	0.121
Proteinuria (Yes/No)	97152864.56 (0- Infinity)	0.993	25.345 (3.539-181.520)	0.001	0.769 (0.366-1.617)	0.489	1.047 (0.546-2.011)	0.889
Hypertension (Yes/No)	2.411 (0.335-17.334)	0.382	3.344 (0.467-23.954)	0.230	0.558 (0.135-2.317)	0.422	2.041 (0.283-14.724)	0.479
Prevalence of CVD (Yes/No)	0.724 (0.451-1.162)	0.181	0.167 (0.043-0.657)	0.010	1.885 (1.013-3.508)	0.045	1.579 (0.957-2.604)	0.074
T_COV_ Prevalence of CVD	–	–	1.788 (1.089-2.934)	0.022	–	–	–	–
Use of RASI (Yes/No)	1.078 (0.605-1.919)	0.799	0.727 (0.474-1.115)	0.144	0.633 (0.316-1.268)	0.197	1.123 (0.585-2.157)	0.727
Use of CCB (Yes/No)	1.046 (0.667-1.641)	0.844	1.735 (1.148-2.624)	0.009	0.478 (0.255-0.894)	0.021	0.718 (0.436-1.183)	0.193
Use of diuretics (Yes/No)	0.975 (0.629-1.512)	0.910	1.178 (0.815-1.704)	0.383	1.492 (0.797-2.794)	0.211	1.087 (0.660-1.788)	0.744

Abbreviations: DKD: diabetic kidney disease; NDKD: non-diabetic kidney disease; SBP: systolic blood pressure; BMI: body mass index; Hb: hemoglobin; Alb: albumin; T_COV_: time-dependent covariates; Scr: serum creatinine; eGFR: estimated glomerular filtration rate; CVD: cardiovascular disease; CKD: chronic kidney disease; RASI: renin-angiotensin system inhibitors; CCB: calcium channel blocker.

The Cox regression model with KRT endpoint as an outcome showed that the increased SBP (HR: 1.012, 95% CI [1.003–1.020], *p* < 0.05), DKD (HR for DKD *vs*. NDKD: 1.604, 95%CI [1.049–2.454], *p* < 0.05), decreased serum Alb (HR:0.943, 95% CI [0.903–0.984], *p* < 0.05), Scr (HR: EXP(2.076–0.892*In(t)), *p* < 0.001), higher CKD stages (HR:6.984, 95% CI [4.185–11.656], *p* < 0.001), presence of proteinuria (HR:8.582, 95% CI [1.163–63.300], *p* < 0.05), prevalence of CVD (HR: EXP(2.813–0.894*In(t)), *p* < 0.001) were independent risk factors for KRT endpoint (Figure 2(B)).

The results of the Cox regression analyses for the all-cause death endpoint showed that the increased age (HR:1.063, 95% CI [1.024–1.104], *p* < 0.05), decreased Hb level (HR: 0.971, 95% CI [0.953–0.989], *p* < 0.05), decreased serum Alb level (HR: 0.928, 95% CI [0.880–0.978], *p* < 0.05) were independent risk factors for all-cause death endpoint (Figure 2(C)).

The results of the Cox regression analyses for CVD events endpoint showed that the increased age (HR: 1.035, 95% CI [1.009–1.062], *p* < 0.05), decreased serum Alb level (HR: 0.937, 95% CI [0.902–0.974], *p* < 0.05) were independent risk factors for CVD events endpoint (Figure 2(D)).

The Cox regression models for 50% eGFR decline, KRT, all-cause death, and CVD events endpoints were all statistically significant (Omnibus test *p* < 0.001).

## 4. Discussion

The global burden of CKD especially DKD is growing which needs to be given much attention by researchers. Early detection of patients who are most likely to progress to kidney complicated endpoints can promote primary care-based treatment to improve the prognosis of CKD. In the present study to compare the long‐term outcome of CKD patients, we found that the cumulative incidence of primary endpoints of DKD is much higher than NDKD patients during the 3-year follow‐up after adjusting for a range of potentially confounding factors by PSM and Cox regression analyses. Meanwhile, the cumulative hazard curves showed that DKD patients exhibit a significantly faster progression in 50% eGFR decline and KRT endpoints. And the DKD patients had a higher risk to progress to 50% eGFR decline (HR: 2.30, 95% CI [1.48–3.58]) and KRT endpoints (HR: 1.64, 95% CI [1.13–2.37]). On the contrary, NDKD patients yielded a lower cumulative incidence of primary endpoints and a longer progression-free survival time compared with DKD patients. Therefore, compared with other etiologies of CKD, patients with DKD have a higher risk to progress to ESRD and require an earlier KRT which imposes substantial health and economic burden on patients and society.

However, there was no significant difference between the two groups in the occurrence of secondary endpoint events including all-cause death and CVD events after PSM. The multivariate Cox regression analyses also showed that DKD is one of the risk factors for 50% eGFR decline and KRT endpoints but not for all-cause death and CVD events after adjusting for other potential confounding factors. It has been established that there is an enhanced inflammation and oxidative stress [12], activated renin-angiotensin system [13], and endothelial dysfunction [14] in DKD status. And these factors are highly associated with CKD progression and adverse complications such as increased mortality and CVD events. Therefore, the possible reason for this discrepancy may lie in the fact that we matched the baseline data such as the prevalence of CVD and hypertension which could influence the incidence of CVD events and all-cause death.

To further investigate the potential predictive factors for CKD endpoints, we performed the multivariate Cox regression analyses and we found that the increased SBP, DKD, decreased serum Alb, higher CKD stages were independent risk factors for 50% eGFR decline endpoint. Additionally, we also found that the increased SBP, DKD, decreased serum Alb, Scr, higher CKD stages, presence of proteinuria, prevalence of CVD were independent risk factors for the KRT endpoint; the increased age, decreased Hb level, decreased serum Alb level were independent risk factors for all-cause death endpoint; the increased age, decreased serum Alb level were independent risk factors for CVD events endpoint. Previous studies have proved that lower serum Alb level is associated with CKD progression [15] and is an important predictor for evaluating the risk and prognosis of CVD events [16]. Our results were also consistent with these findings. We proved that lower serum Alb is an independent risk factor for 50% eGFR decline, KRT, all-cause death, and CVD events endpoints after adjusting for other confounding factors. Meanwhile, we also proved that the decreased Hb level is also an independent risk factor for all-cause death endpoint. Just similar to the serum Alb indicator, a meta‐analysis indicated that Hb is also a useful biomarker for malnutrition [17]. It’s widely recognized that malnutrition implicates the existence of inflammation, which is also known as the malnutrition-inflammation-atherosclerosis (MIA) syndrome [18]. Furthermore, studies have shown that MIA is closely associated with increased mortality of CKD patients [19,20]. Based on our findings and these past researches, we can state that malnutrition status is a strong predictive factor for the poor prognosis of CKD patients. Therefore, attention should be paid to the CKD patients with anemia or hypoalbuminemia to prevent malnutrition-associated complications.

We also found that the increased SBP, DKD, higher CKD stage were risk factors for not only 50% eGFR decline endpoint but also KRT endpoint. In summary, these indicators were closely associated with the deterioration of kidney function. A previous study revealed that approximately 60% to 90% of CKD patients may be combined with hypertension [21], we also proved that the prevalence of hypertension is high both in DKD and NDKD groups (98.5% in the DKD group and 94.9% in the NDKD group after PSM). Therefore, hypertension is a common comorbidity in CKD patients. A prospective cohort Chronic Renal Insufficiency Cohort (CRIC) Study from Americans showed that SBP ≥140 mmHg is associated with increased risk of CKD progression [22]. This finding is in accordance with our present results from the East Asian population. Despite sustained hypertension can lead to worsening kidney function, progressive decline in kidney function can conversely lead to worsening blood pressure control [21]. Moreover, intensive blood pressure-lowering therapy has been shown to reduce adverse CVD events and all-cause mortality in CKD patients [21,23]. Our present study showed that blood pressure-lowering therapy may be also beneficial in retarding the deterioration of kidney function. An increasing body of evidence supports the effect of intensive glucose control in reducing the risk of adverse renal events [23]. Our present study also showed that DKD is an independent risk factor for 50% eGFR decline and KRT endpoints. Therefore, excellent long-term control of glucose may not only delay the progression of DKD but also improve the kidney endpoint events. In the current study, we also found that the elevated Scr, the presence of proteinuria, and CVD were predictive factors for KRT events. Undoubtedly, Scr is a commonly used clinical indicator for evaluating kidney function. However, it should be noted that the level of Scr may be influenced by muscle mass [24] which is also an indicator of nutritional status [25]. In the present study, eGFR was calculated using Scr and age developed by the Japanese Society of Nephrology [7,9]. Thus, the detection of Scr should be combined with the evaluation of nutritional status to better comprehensively evaluate kidney function. Proteinuria is a marker of kidney damage [26] and doubling of proteinuria is associated with a higher risk of subsequent kidney failure and mortality [24]. We also proved that the presence of proteinuria is an independent predictive factor for KRT events. Therefore, the dynamic detection of proteinuria in the course of CKD is helpful to evaluate the prognosis of CKD patients and identify high-risk populations. All of these indicators are easily identifiable and can be used for predicting the prognosis of CKD patients. By early identifying these risk factors, appropriate interventions can be exerted to improve the prognosis and reduce the disease burden of CKD patients.

In conclusion, this prospective multicenter cohort study based on the East Asian population suggests that DKD patients remain at a higher risk to develop 50% eGFR decline and KRT endpoints even after matching for the baseline characteristics. The increased SBP, DKD, decreased serum Alb, higher CKD stages were independent risk factors for 50% eGFR decline endpoint. The increased SBP, DKD, decreased serum Alb, Scr, higher CKD stages, presence of proteinuria, prevalence of CVD were independent risk factors for the KRT endpoint; the increased age, decreased Hb level, decreased serum Alb level were independent risk factors for all-cause death endpoint; the increased age, decreased serum Alb level were independent risk factors for CVD events endpoint. Attention should be paid to the high-risk population and early preventive or therapeutic interventions should be taken to delay the development of CKD complications, thereby improving the prognosis and reducing the disease burden of CKD patients.

The present study exhibits some limitations. Despite a PSM design has been used to assemble a balanced cohort, potential biases may exist in unmeasured or unknown factors. Next, the CKD-ROUTE study is restricted to Japanese patients in East Asia, hence its generalizability to other races may be limited. In recent years, the application of sodium-glucose cotransporter 2 (SGLT2) inhibitors, glucagon-like peptide-1 (GLP-1) receptor agonists, and dipeptidyl peptidase-4 (DPP-4) inhibitors have largely improved the prognosis of DKD patients, therefore further studies are needed to investigate the outcomes between the two groups after treatment.

## Supplementary Material

Supplemental Material

Supplemental Material

Supplemental Material

Supplemental Material

## Data Availability

The readers can access the data that support the findings of this study through the Dryad repository (DOI: 10.5061/dryad.kq23s).

## Geolocation information

East Asia
