# Supplementary figures and images for: Prognosis and risk factors of chronic kidney disease progression in patients with diabetic kidney disease and non-diabetic kidney disease: a prospective cohort CKD-ROUTE study
Source: Ren Fail. 2022 Aug 8;44(1):1309–18. doi: 10.1080/0886022X.2022.2106872 (PMC9361770; doi:10.1080/0886022X.2022.2106872)

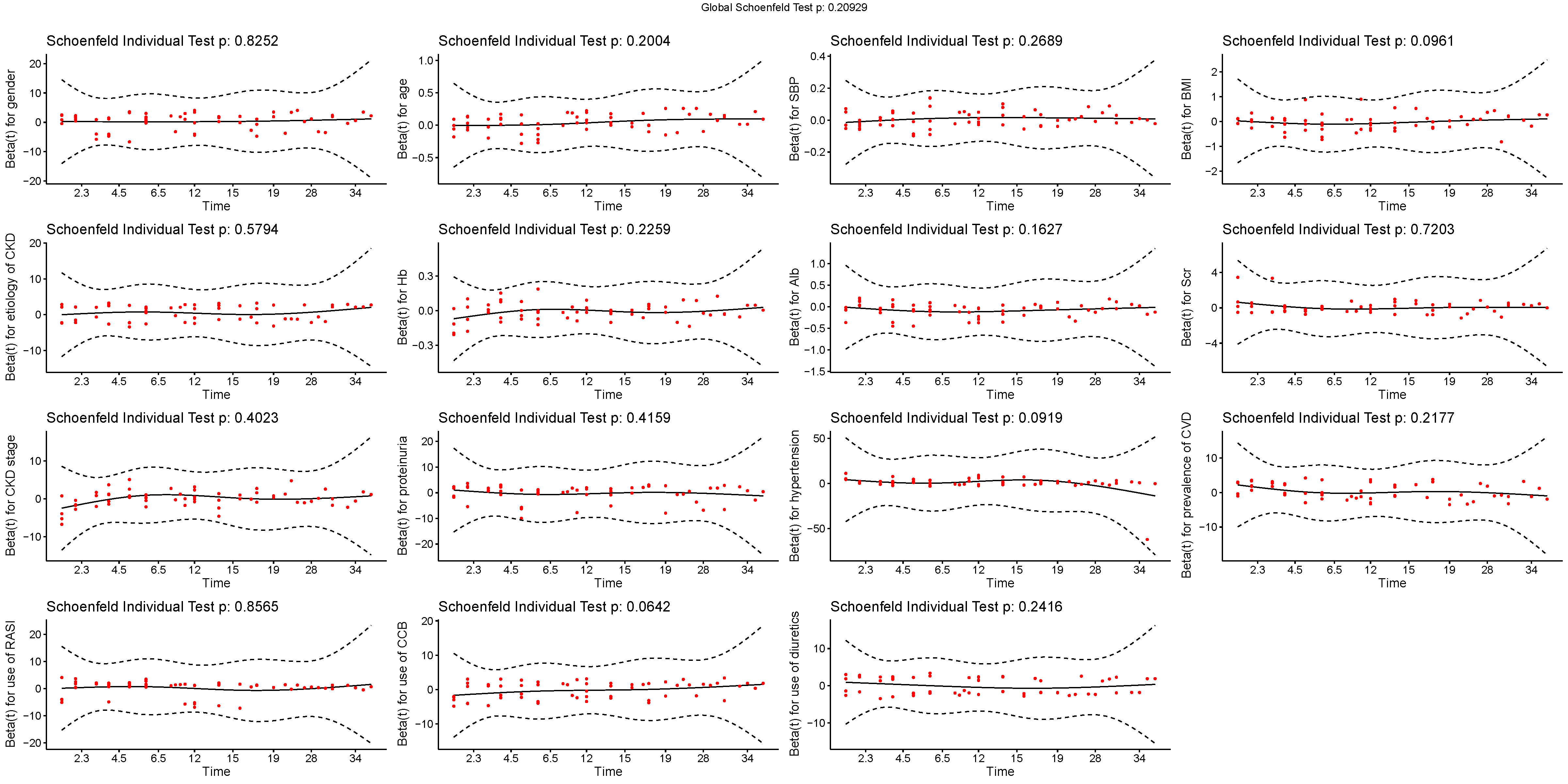

Supplement: Supplemental Material [file IRNF_A_2106872_SM7460.tiff]

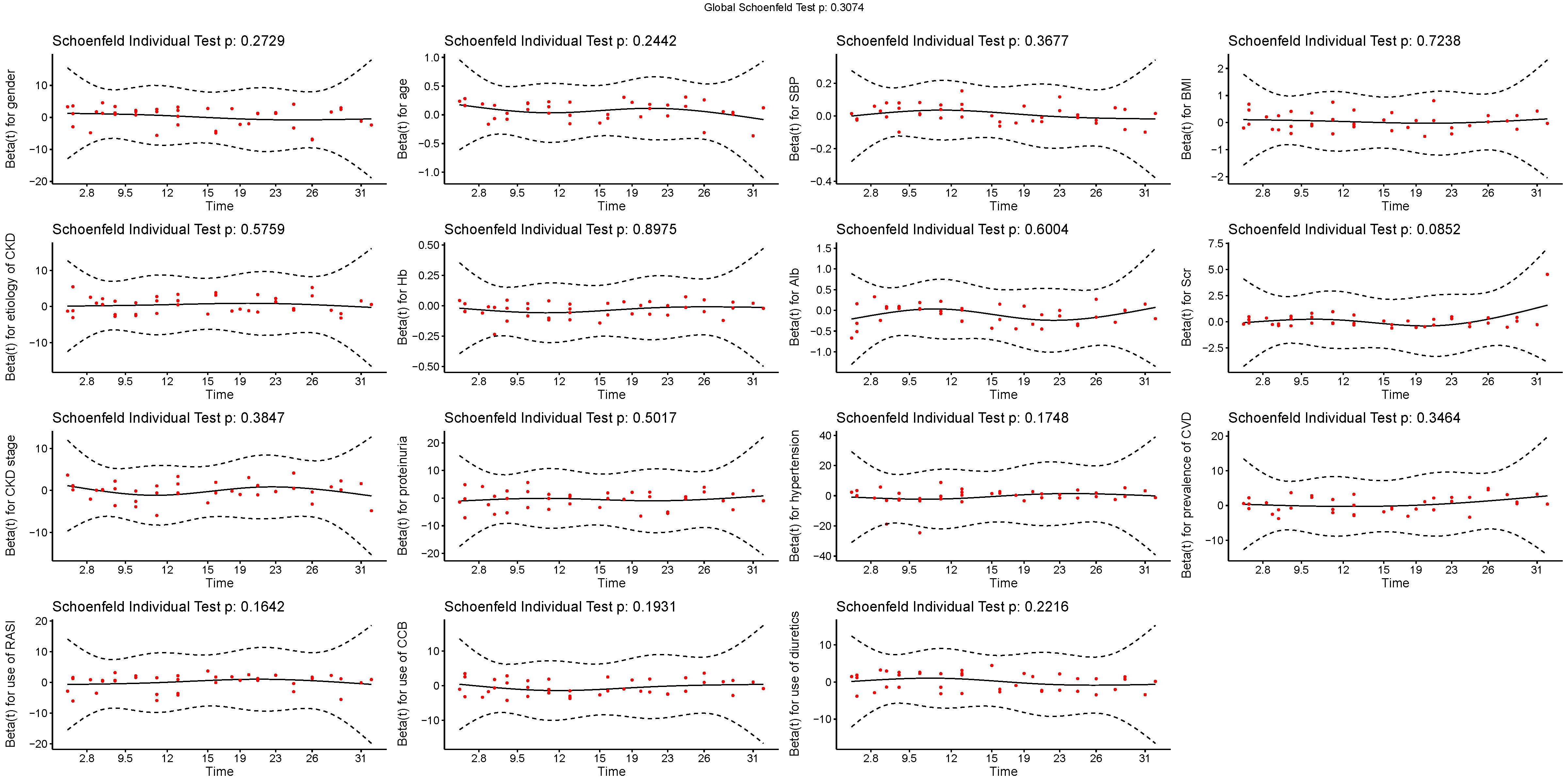

Supplement: Supplemental Material [file IRNF_A_2106872_SM7458.tiff]

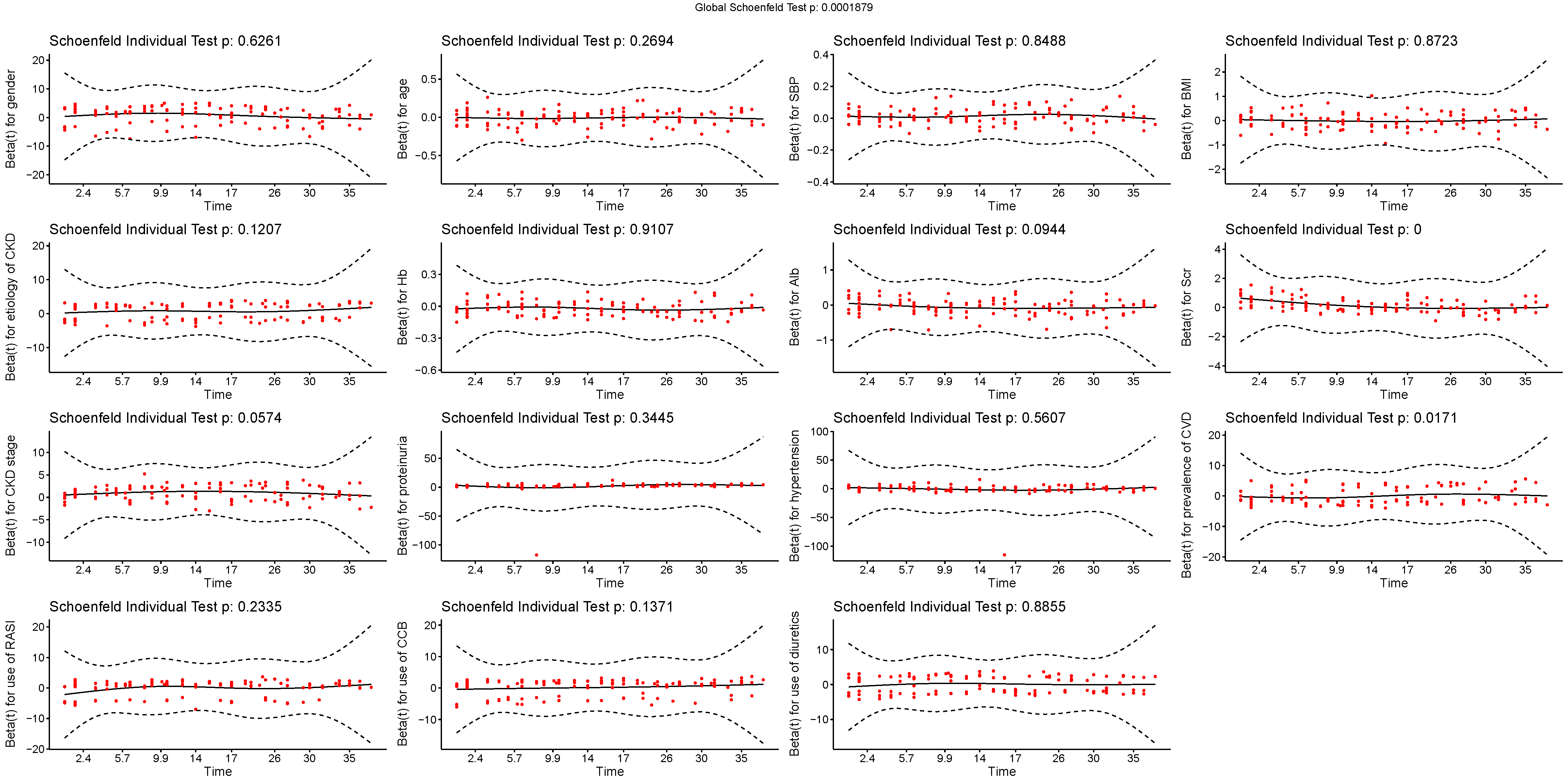

Supplement: Supplemental Material [file IRNF_A_2106872_SM7456.tiff]

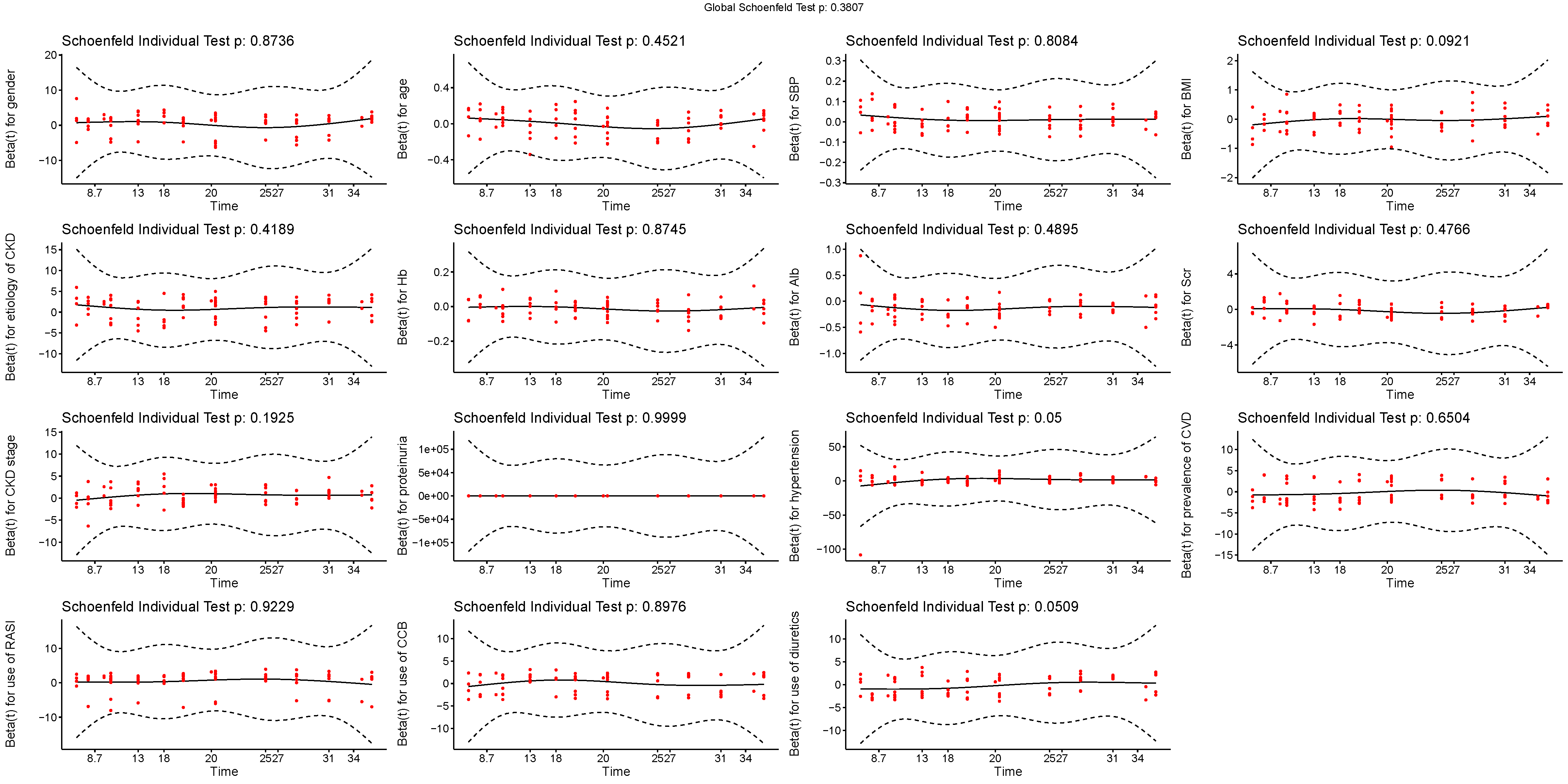

Supplement: Supplemental Material [file IRNF_A_2106872_SM7452.tif]
